# Ascorbic acid metabolites are involved in intraocular pressure control in the general population

**DOI:** 10.1016/j.redox.2018.10.004

**Published:** 2018-10-13

**Authors:** Pirro G. Hysi, Anthony P. Khawaja, Cristina Menni, Bani Tamraz, Nick Wareham, Kay-Tee Khaw, Paul J. Foster, Leslie Z. Benet, Tim D. Spector, Chris J. Hammond

**Affiliations:** a Section of Academic Ophthalmology, Faculty of Life Sciences and Medicine, Kings' College London, UK; b King’s College London Department of Twin Research and Genetic Epidemiology, London, UK; c Department of Public Health & Primary Care, University of Cambridge, UK; d NIHR Biomedical Research Centre for Ophthalmology, Moorfields Eye Hospital and University College London, UK; e University of California, San Francisco, School of Pharmacy, San Francisco, CA, USA; f University of California, San Francisco, School of Pharmacy, Department of Bioengineering and Therapeutic Sciences, San Francisco, CA, USA

**Keywords:** Ascorbate metabolism, Intraocular pressure, Multi-omics

## Abstract

Elevated intraocular pressure (IOP) is an important risk factor for glaucoma. Mechanisms involved in its homeostasis are not well understood, but associations between metabolic factors and IOP have been reported. To investigate the relationship between levels of circulating metabolites and IOP, we performed a metabolome-wide association using a machine learning algorithm, and then employing Mendelian Randomization models to further explore the strength and directionality of effect of the metabolites on IOP. We show that O-methylascorbate, a circulating Vitamin C metabolite, has a significant IOP-lowering effect, consistent with previous knowledge of the anti-hypertensive and anti-oxidative role of ascorbate compounds. These results enhance understanding of IOP control and may potentially benefit future IOP treatment and reduce vision loss from glaucoma.

## Highlights

• Vitamin C and its metabolites are highly concentrated in human brain and eye tissues.
• Multi-omics analysis finds evidence for association between ascorbic acid metabolism and intraocular pressure.
• O-methylascorbate lowers intraocular pressure in the general population.
• O-methylascorbate's role in intraocular pressure regulation is likely mediated by its anti-photooxidative properties.

## 1 Introduction

Glaucoma is a leading cause of irreversible blindness and an important public health concern. Better understanding of its pathophysiology is important because it might lead to earlier detection and improved management strategies. Glaucoma and intraocular pressure (IOP) are tightly correlated genetically and epidemiologically [1], but our understanding of cell and tissue-level processes underlying elevated IOP and glaucoma are not well understood.

The eyes share cellular metabolic pathways and physiological mechanisms with other organs and tissues. Genes associated with IOP and POAG are involved, among others, in systemic lipid metabolism [1], [2], lysosomal endocytosis [3] and angiogenesis [1], [4]. Additionally, both IOP [5] and POAG [6] are strongly associated with the components of the metabolic syndrome (hyperglycemia, hyperlipidemia and high systemic blood pressure).

The purposes of this work were to investigate the relationship between circulating metabolites and IOP, by performing a metabolome-wide association study, and to examine the causality direction of such relationships, using Mendelian Randomization (MR) in independent population-based cohorts.

## 2 Materials and methods

### 2.1 Study design

This work followed two stages. First, associations between circulating metabolite levels of individual metabolites and IOP were identified in a population-based cohort (TwinsUK). Subsequently, Mendelian Randomization (MR) analyses in two independent populations were used to validate the relationship between circulating metabolite levels and IOP and assess causality.

### 2.2 Populations and subjects

#### 2.2.1 TwinsUK

This is a volunteer cohort recruited from the general population in the United Kingdom [7]. Included in this study are 1763 adults (684 twin pairs and 395 singletons), for whom both metabolite levels and eye measurements including IOP were available.

The IOP measurements were taken using a non-contact air-puff tonometer (Ocular Response Analyzer, ORA, Reichert, Buffalo, NY). The mean IOP was calculated from four readings (two from each eye). Subjects who were receiving IOP-lowering medications or had IOP-altering surgery were excluded from the analyses.

#### 2.2.2 UK Biobank

UK Biobank is a large multisite cohort study of UK residents aged 40–69 years. Participants’ IOP was measured once per eye using ORA. Participants with a history of eye surgery or injury and with IOP measurements in the top and bottom 0.5 percentiles were excluded. The pre-treatment IOP of the 1571 participants under IOP-lowering medication was imputed as 130% of the measured mean IOP to allow for medication effect as previously recommended [8], [9], [10]. IOP was calculated as the mean of right and left eye ORA IOPcc parameter values for each participant. Effect size estimations for subsequent MR analyses were extracted from the results of association between genotypes and IOP, described elsewhere [1].

#### 2.2.3 EPIC-Norfolk

EPIC-Norfolk is one of the UK arms of the European Prospective Investigation into Cancer (EPIC) study [11]. Detailed ophthalmic assessments using ORA and genotypes were available for 8623 participants. The quality control, inclusion and exclusion criteria, QC steps and linear regression methods that were used to generate results, are described in detail elsewhere [1].

#### 2.2.4 Ethical approvals

This study was conducted in accordance with the principles of the Declaration of Helsinki and the Research Governance Framework for Health and Social Care. All participants gave informed consent after appropriate ethics committee approval: Guy's and Saint Thomas (GSTT) for the TwinsUK, the North-West Research Ethics Committee for the UK Biobank and the Norfolk Local Research Ethics Committee and East Norfolk & Waveney NHS Research Governance Committee for the EPIC-Norfolk participants.

### 2.3 Metabolite measurements

Non-targeted metabolite detection and quantification was conducted using the platform provided by Metabolon Inc. (Durham, USA) on fasting plasma samples as previously described [12]. Quality control steps for the 529 measured metabolites are reported elsewhere [12], and they included batch-effect data normalization, outlier (>4 SD) removal and subsequently inverse-normalization [13]. Only 313 metabolites that were measured in at least 90% of subjects were included in analyses.

### 2.4 Statistical analyses

#### 2.4.1 Random Forest analysis of metabolite effects on IOP

Although metabolites may be univariably associated with genetic factors, their ratios and other higher-order forms of interaction between more than one metabolite have physiologic relevance and are under tight genetic control [12]. Simple linear regression models therefore are incapable of fully modelling these interactions. Here, we employed a Random Forest [14] machine learning technique (RF) which agnostically identifies the metabolites that are most influential over an outcome, regardless of the specific model through which the effect is mediated. These models implicitly capture higher order interactions between variables [15]. We used RF to rank all available metabolites according to the Breiman-Cutler “VIMP” values [14]. Importance ranking has no associated probabilities, or formal thresholds of significance, nor any need for multiple-testing correction as long as all variables are tested jointly at the same time. To control for bias from predictor variables’ (metabolites) differing variances [16], all variables were standard inverse-normalized as previously described [13]. The ‘*mtry*’ parameter was fine-tuned to minimize the out-of-bag errors. The parameters of the RF analyses were set as *nTree* = 10,000 and *maxNodes* = 10 and *mtry* = 140. The models also included confoundants such as ages at IOP measurement and when the blood samples were drawn, body height and weight, but only the relative importance of metabolites over IOP measurements is being reported. Specifically, the model included a mixed model adjustment term to address the family relationships among the participants of the TwinsUK cohort.

Analyses reported here were conducted using all available metabolites that passed QC; analyses on subsets of unrelated metabolites (not shown) did not produce fundamental alterations in the importance ranking of the metabolites representing their clusters. Analyses were run in the ‘randomForestSRC’ package, version 2.5.1 in R 3.4.1(www.cran.r-project.org).

#### 2.4.2 Mendelian randomization comparisons of genetic effects

We aimed to validate the findings and assess causality for the most important metabolite identified in the RF analysis stage, through an MR model. We used as instrumental variables (IV) SNPs that associated with plasma metabolite levels (exposure) on IOP (outcome).

Effects of genetic variants over metabolite levels were obtained from a published study [12]. We used SNPs that showed association at either GWAS-significant (p < 10^−08^), but also at suggestive levels (p < 10^−06^) in the final published joint meta-analyses [12]. Estimates of effect sizes and standard errors for the association between the selected SNPs and metabolites were from the Kooperative Gesundheitsforschung in der Region Augsburg (KORA) cohort and were obtained from previously published reports [17]. Only SNPs that were independent (on different chromosomes or at least 4 million base pairs apart and r^2^ < 0.1) were used for the analyses. Estimated effect sizes and standard errors for association of the SNPs with IOP were obtained from a GWAS of 103,382 European participants of the UK Biobank and separately from 6595 participants in the EPIC-Norfolk study, as reported elsewhere [1].

Three MR methods were used: inverse variance weighted median, inverse-variance weighted and MR-Egger. These analyses are usually interpreted together to jointly evaluate the relationship between exposure and outcome [18], [19] and don’t require multiple testing correction. The MR-Egger regression test intercept evaluates evidence for directional pleiotropy; intercepts significantly different from the origin suggest directional pleiotropy, where the underlying Instrument Strength Independent of Direct Effect (InSIDE) assumption may not be satisfied [20]. Analyses were performed using the ‘MendelianRandomization’ R package [21].

## 3 Results

### 3.1 O-methylascorbate levels are associated with IOP

We studied the plasma levels of 313 metabolites in the dataset of 1772 TwinsUK participants, for whom IOP measurements were also available. The main demographic and clinical characteristics of the sample are summarized in Table 1.

**Table 1:** **Main demographic and clinical characteristics of the participating cohorts**. Mean and standard deviations are given for each parameter; missing values (“NA”) are used for variables not measured in a particular cohort.

	TwinsUK		UK Biobank		EPIC-Norfolk	
Variable	Mean	Standard Deviation	Mean	Standard Deviation	Mean	Standard Deviation
Age at the time IOP was measured (years)	55	9.13	54.4	7.8	68.8	8
Age when blood samples were taken (years)	58.4	9.87	NA	NA	NA	NA
Sex (women: men)	1755: 8	NA	55,103: 48,279	NA	3725:3059	NA
Mean intraocular pressure (mmHg)	15.6	3.18	16.1	3.5	16.8	3.6
Central corneal thickness (µm)	544.9	39.58	NA	NA	–	–
Weight (kg)	69.45	13.6	77.98	15.9	74.2	14.1
Height (cm)	161.8	6.16	170.1	9.4	166.4	9.1

**Fig. 1:**
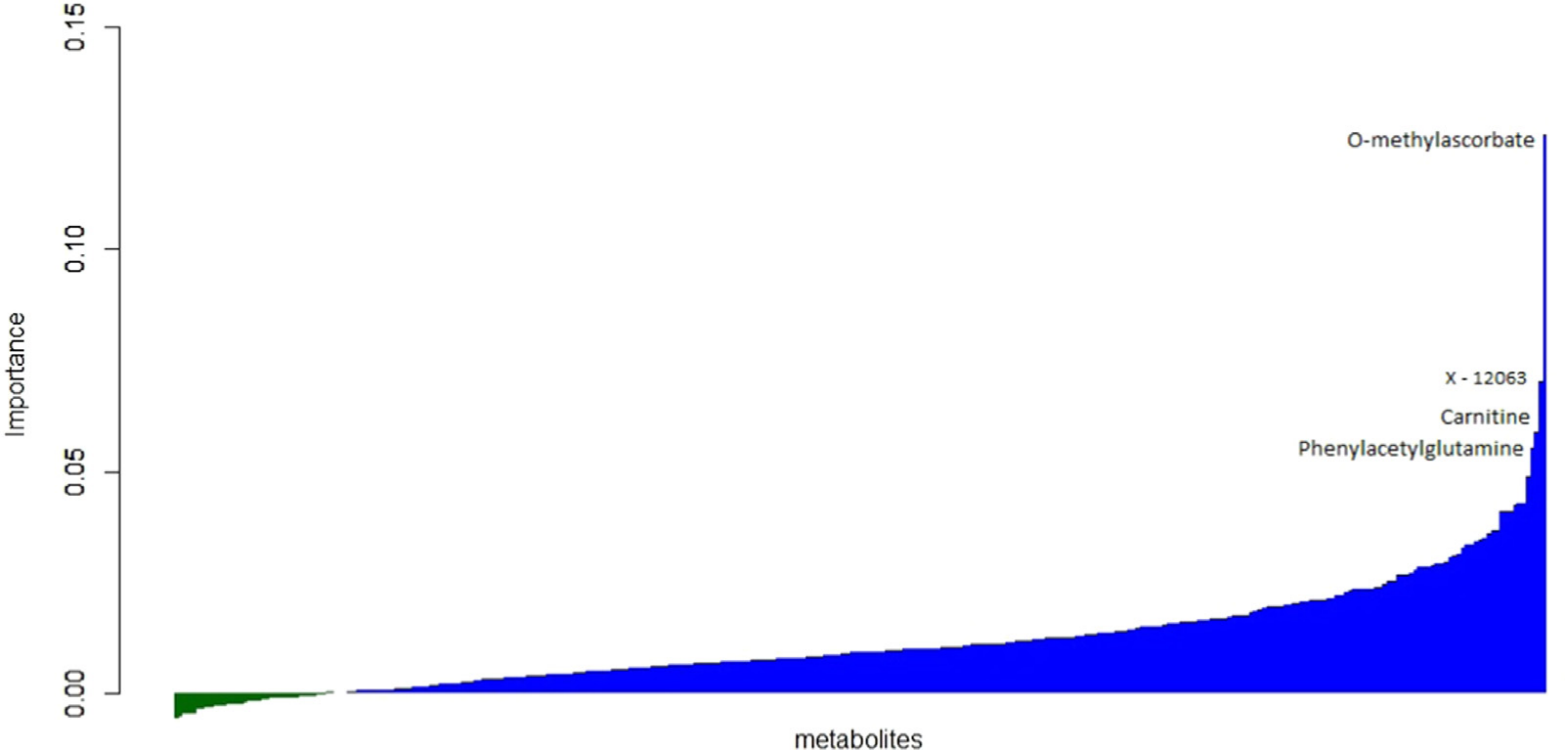
Plot of the VIMP parameter (relative importance) of the associations with IOP of 313 metabolite variables tested in the Random Forest analysis. The metabolites with highest importance are labeled (X- 12063 uncharacterized metabolite, identity unknown).

A random forest (RF) analysis ordered metabolites according to the importance of their association with IOP (Fig. 1). The highest-ranking metabolite in order of importance was O-methylascorbate [22]. This is a known metabolic product of the L-ascorbic acid (Vitamin C) [23]. Polymorphic changes of the sequences of the *COMT*, but also *KLF12*, *SIL1*, *FDFT1* and *PPPC5* genes are associated with O-methylascorbate levels [12]. The second ranking metabolite from the RF analysis was alpha-hydroxyvalerate, an amino acid metabolite. High in the rankings (Supplementary Table 1) were also carnitine, involved in lipid transfer across the mitochondrial membrane [24] and phenylacetylglutamine, a metabolite of glutamate, an antioxidative stress marker.

### 3.2 O-methylascorbate reduces IOP in independent populations

**Fig. 2:**
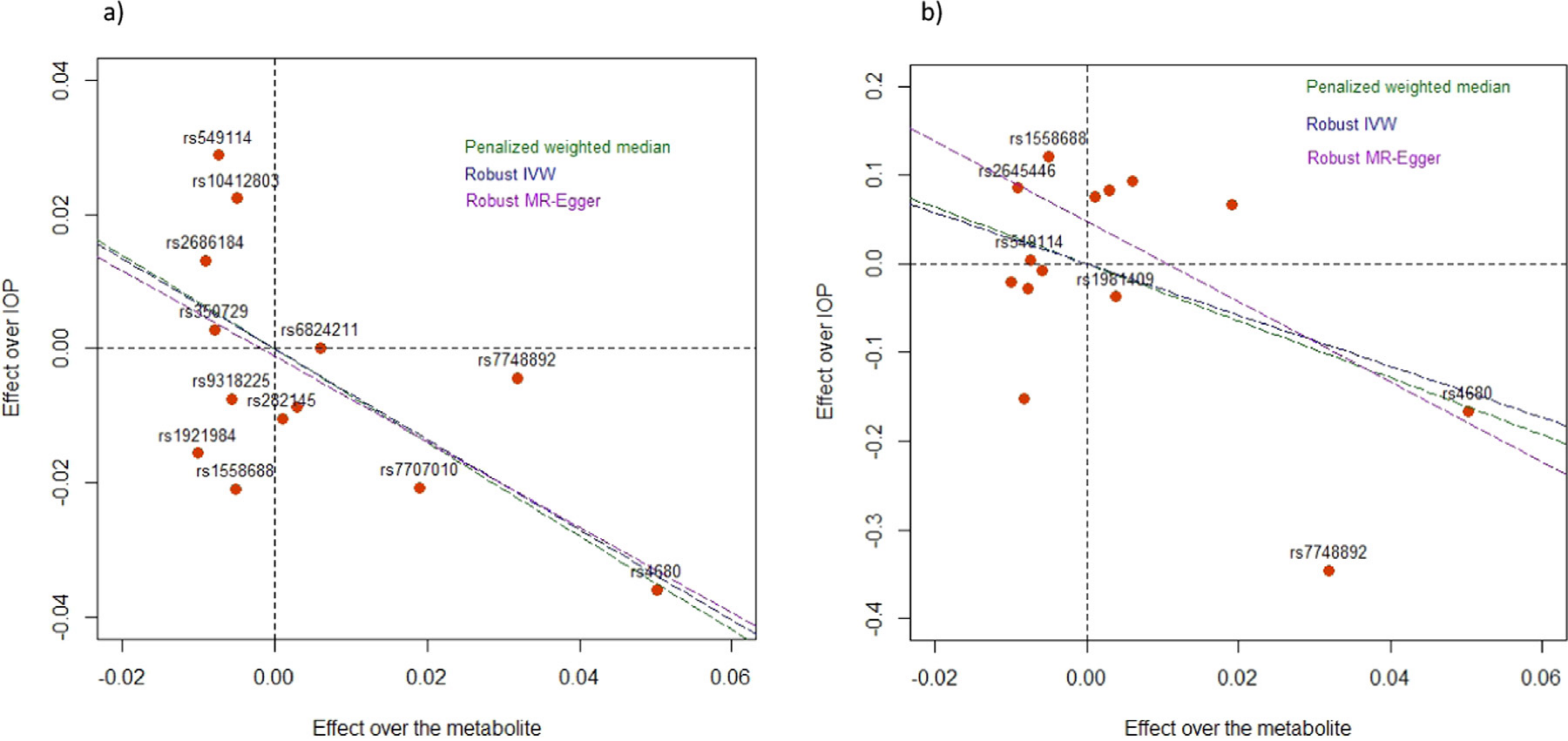
Relationship of observed effect sizes of the instrumental variable SNP on IOP in the UK Biobank (a) and the EPIC-Norfolk (b) cohorts with the effect sizes of the same SNPs on O-methylascorbate levels in the KORA population. The lines represent the regression slopes for the different models, as specified in the legend.

We followed up on the highest-ranking metabolite from the RF analysis. To validate results, we explored the relationship between O-methylascorbate and IOP in two independent populations. We used as genetic instruments single nucleotide polymorphisms (SNPs) that were significantly associated with O-methylascorbate levels in the KORA cohort [12], and examined their association with IOP, initially, in the UK Biobank cohort (Supplementary Table 2). A Mendelian Randomization (MR) model found a significant relationship between exposure (the O-methylascorbate levels) and IOP. All three models (Table 2, Fig. 2**a**) showed a statistically significant inverse relationship between the circulating levels of this metabolite and IOP (weighted median p = 0.02, robust IVW p = 2.4 ×10^−10^ and RM-Egger p = 3.33 × 10^−06^). There was no statistical evidence of pleiotropy (MR-Egger Intercept = 0.00). We further used the same instrumental variables(IVs) to build a second MR model in another independent dataset. The MR results in the EPIC-Norfolk dataset were consistent with the results obtained in the UK Biobank, with statistically significant effects of O-methylascorbate on IOP (Table 2 and Fig. 2**b**).

**Table 2:** **Mendelian Randomization (MR) study results for IOP in the UK Biobank and EPIC-Norfolk cohorts**. For each of the three methods used, the β estimate, standard errors (SE) and associated p-values are reported. The Penalized robust MR-Egger intercept is not a MR model, but if different from 0 would provide evidence of directional pleiotropy and potential violation of the instrumental variable assumptions.

	UK Biobank			EPIC		
Method	Beta	SE	p-value	Beta	SE	p-value
Penalized weighted median	−0.696	0.304	0.022	−3.219	1.371	0.019
Robust inverse-variance weighted	−0.674	0.106	2.04 × 10^−10^	−2.891	0.678	2.5 × 10^−05^
Robust MR-Egger	−0.637	0.137	3.33 × 10^−06^	−4.536	0.689	4.6 × 10^–11^
Penalized robust MR-Egger (Intercept)	−0.001	0.006	0.855	0.048	0.026	0.071

To further exclude pleiotropy, we reversed our MR models to use IOP as the putative risk, SNPs significantly associated with IOP, described elsewhere [1] (Supplementary Table 3) as IVs, IOP as exposure and O-methylascorbate as the outcome of interest. In contrast to the previous results, none of the tests were statistically significant (Supplementary Table 4), which further suggests that O-methylascorbate levels causally affect IOP and not vice versa.

## 4 Discussion

Here we report, for the first time, that O-methylascorbate, a Vitamin C metabolite, is part of metabolic mechanisms that control IOP in the general population. Previous works suggested that levels of Vitamin C are reportedly inversely correlated with systemic [25] and pulmonary [26], [27] blood pressure, as well as IOP [28], [29]. Although it also enhances endothelial function [30], much of its anti-hypertensive effects are likely mediated by its powerful antioxidative properties, which provide protection from the radical oxygen species [31].

Photooxidative stress in the eye leads to trabecular meshwork degradation [32], elevated IOP due to increased aqueous outflow resistance [33] and ultimately glaucoma [34]. Vitamin C is highly concentrated in the aqueous humor and forms the first line of defense against free radicals in the eyes [35]. The O-methylascorbate, a naturally occurring metabolite, is less cytotoxic [36] and has a strong reductive capacity against photooxidative stress [37].

Our study combined metabolomic and genetic data to identify metabolic processes that modulate IOP in healthy populations. Our MR results shows that genetic factors that raise O-methylascorbate levels are associated with lower IOP. For example, the rs4680 G allele leads to higher COMT activity [38], and increased enzymatic conversion of Vitamin C into O-methylascorbate [12]. This variant is also associated with lower IOP, likely through the antioxidant properties of its enzymatic reaction product. The correlations of effects observed over several genes that independently control O-methylascorbate levels suggests that O-methylascorbate effect on IOP is real and not the result of confounding.

Several considerations are needed for the correct interpretation of these findings. First, O-methylascorbate effects over IOP homeostasis are likely modest and not deterministic. The metabolome platform that we used, only provides semi-quantitative results, but its standard-normalized output may be used to assess the strength of statistical associations, but not reliable effect size estimation. Second, the metabolomic platform we used only assesses a fraction of the metabolites present in complex organisms and could have overlooked metabolites equally or more relevant to IOP homeostasis. Third, the TwinsUK discovery cohort had power limitations and is almost exclusively female, while associations between oxidative biomarkers and glaucoma are reportedly stronger in men [29]. Finally, while the causal inference statistical methods suggest a causative role for O-methylascorbate in IOP, MR methodologies critically rely on several assumptions, whose violations would change the interpretation of causality [19]. Until further experimental confirmation, the relationship of O- methylascorbate with IOP is simply probabilistic.

Our study demonstrates that Vitamin C metabolism is involved in the control of intraocular pressure. These findings provide an additional insight into the role antioxidative stress-related mechanisms in intraocular, and maybe blood pressure homeostasis. Further work will be necessary to establish the exact mechanisms of pressure reduction via ascorbate metabolites and establish whether these mechanisms may have any role for the clinical management of IOP or glaucoma.
