# Supplementary material for: Ascorbic acid metabolites are involved in intraocular pressure control in the general population
Source: Redox Biol. 2018 Oct 13;20:349–53. doi: 10.1016/j.redox.2018.10.004 (PMC6223183; doi:10.1016/j.redox.2018.10.004)
Supplement: Supplementary file 1 — Supplementary material [file mmc1.docx]

**Supplementary Materials**

**Contents**

[**Acknowledgements & Author Contributions** 2](#_Toc524016100)

[**Supplementary Table 1. Importance of the metabolite variables in random forest models for IOP** 3](#_Toc524016101)

[**Supplementary Table 2. List of SNP instrumental variables used for MR analyses of O-methylascorbate on IOP** 4](#_Toc524016102)

[**Supplementary Table 3. Instrumental variables used for the MR analysis of the putative effects of IOP on levels of O-methylascorbate** 5](#_Toc524016103)

[***Supplementary Table 4. Mendelian Randomization tests using IOP-associated SNPs from the genetic association analyses in the UK Biobank as instruments over O-Methylascorbate (KORA)*** 7](#_Toc524016104)

# **Acknowledgements & Author Contributions**

EPIC-Norfolk infrastructure and core functions are supported by grants from the Medical Research Council (G1000143) and Cancer Research UK (C864/A14136). The clinic for the third health examination was funded by Research into Ageing (262). Genotyping was funded by the Medical Research Council (MC_PC_13048). We thank all staff from the MRC Epidemiology laboratory team for the preparation and quality control of DNA samples. Mr Khawaja is supported by a Moorfields Eye Charity fellowship. Professor Foster has received additional support from the Richard Desmond Charitable Trust (via Fight for Sight) and the Department for Health through the award made by the National Institute for Health Research to Moorfields Eye Hospital and the UCL Institute of Ophthalmology for a specialist Biomedical Research Centre for Ophthalmology.

TwinsUK is funded by the Wellcome Trust, Medical Research Council, European Union, the National Institute for Health Research (NIHR) – funded BioResource, Clinical Research Facility and Biomedical Research Centre based at Guy’s and St. Thomas’ NHS Foundation Trust in partnership with King’s College London. CH and PH acknowledge the support from the TFC Frost Charitable Trust.

P.G.H. participated in study design and ran the statistical analyses and helped write the manuscript. APK made available data and ran the analyses in the EPIC-Norfolk Cohort, C.M. was responsible for the quality control of the TwinsUK metabolite dataset, B.T. and L.Z.B. helped write the manuscript. NW and KTK made available data from the EPIC-Norfolk study, P.J.F. contributed to the availability of UK Biobank eye data and helped write the manuscript, T.D.S. made TwinsUK metabolite data available and participated in designing metabolite work, C.J.H. participated in study design, made available TwinsUK phenotypic information and helped write the manuscript.

**Supplementary Table 1. Importance of the metabolite variables in random forest models for IOP.** The metabolite ID is the unique identifier of the metabolite in the Metabolon platform. The field “Metabolite” reports the name of the metabolite (when the identity is known). Metabolite names starting with “X” are not fully characterized. “Super” and “Sub-pathways” are larger or smaller branch physiological pathways in which the metabolites are involved.

| **METABOLITE ID** | **METABOLITE** | **SUPER PATHWAY** | **SUB PATHWAY** | **VIMP IMPORTANCE** |
| --- | --- | --- | --- | --- |
| M32910 | O-methylascorbate | --- | --- | 0.126 |
| M33415 | X - 12063 | Insulin sensitivity | --- | 0.070 |
| M15500 | Carnitine | Lipid | Carnitine metabolism | 0.059 |
| M35126 | Phenylacetylglutamine | Amino acid | Phenylalanine & tyrosine metabolism | 0.055 |
| M22032 | X – 08766 | --- | --- | 0.049 |
| M33477 | Erythronate | Carbohydrate | Amino-sugars metabolism | 0.043 |
| M33937 | Alpha-hydroxyisovalerate | Amino acid | Valine, leucine and isoleucine metabolism | 0.043 |
| M01585 | N-acetylalanine |  |  | 0.042 |
| M01604 | Urate | Nucleotide | Purine metabolism, urate metabolism | 0.041 |
| M36808 | Dimethylarginine | Amino acid | Urea cycle; arginine-, proline-, metabolism | 0.041 |

**Supplementary Table 2. List of SNP instrumental variables used for MR analyses of O-methylascorbate on IOP**. The table shows the effect sizes over IOP (calculated in the UK Biobank, N=103,382) of genetic instruments selected based on the significance of their association in a meta-analysis of two populations (shown under “Meta-Analysis p.val” in this table). The original unbiased effect sizes were extracted from the estimates calculated from meta-analyses KORA participants only(9) (shown under “Beta (KORA)” and “SE (KORA)” in this table).

|  |  |  |  |  | SNP association with IOP in UK Biobank | | | Association with O-methylascorbate in KORA | | |
| --- | --- | --- | --- | --- | --- | --- | --- | --- | --- | --- |
| SNP | Chr. | Pos. (Build 37) | Ref. Allele | Other Allele | BETA | SE | P | Beta (KORA) | SE (KORA) | Meta-Analysis p.val |
| rs549114 | 1 | 94534354 | G | A | 0.0288 | 0.0161 | 0.073 | -0.007 | 0.004 | 7.57E-07 |
| rs350729 | 2 | 52983773 | T | G | 0.0029 | 0.0165 | 0.862 | -0.008 | 0.004 | 6.90E-08 |
| rs1921984 | 2 | 217830664 | T | C | -0.0156 | 0.0155 | 0.316 | -0.010 | 0.003 | 9.00E-07 |
| rs6824211 | 4 | 186499013 | G | A | 0.0001 | 0.0165 | 0.993 | 0.006 | 0.004 | 2.16E-07 |
| rs7707010 | 5 | 138444877 | A | G | -0.0207 | 0.0380 | 0.586 | 0.019 | 0.009 | 9.61E-09 |
| rs7748892 | 6 | 137003278 | A | G | -0.0044 | 0.0426 | 0.918 | 0.032 | 0.010 | 4.30E-07 |
| rs1558688 | 7 | 36915185 | T | C | -0.0209 | 0.0167 | 0.210 | -0.005 | 0.004 | 4.34E-07 |
| rs2645446 | 8 | 11649069 | G | C | 0.0129 | 0.0154 | 0.402 | -0.009 | 0.003 | 1.74E-07 |
| rs1887346 | 13 | 74493002 | G | A | 0.0011 | 0.0170 | 0.946 | -0.008 | 0.004 | 6.45E-07 |
| rs1424092 | 16 | 75810140 | G | T | -0.0087 | 0.0158 | 0.580 | 0.003 | 0.004 | 1.78E-07 |
| rs741233 | 19 | 46894786 | C | T | 0.0111 | 0.0164 | 0.499 | -0.006 | 0.004 | 2.92E-07 |
| rs282145 | 20 | 551921 | T | C | -0.0104 | 0.0160 | 0.516 | 0.001 | 0.004 | 7.18E-07 |
| rs4680 | 22 | 19951271 | G | A | -0.0359 | 0.0152 | 0.018 | 0.050 | 0.003 | 4.66E-178 |

**Supplementary Table 3. Instrumental variables used for the MR analysis of the putative effects of IOP on levels of O-methylascorbate**. The labels “IOP.Beta”, “IOP.SE”, “Metab.Beta” and “Metab.SE” refer to the linear regression slopes and their standard errors from the association analyses with IOP (in the UK Biobank) and O-methylascorbate levels, EA is the effect allele, or the reference for which the effects are reported, NEA is the non-reference allele (in the KORA cohort).

| **SNP** | **CHR** | **Pos37** | **NEA** | **EA** | **IOP.Beta** | **IOP.SE** | **Metab.Beta** | **Metab.SE** |
| --- | --- | --- | --- | --- | --- | --- | --- | --- |
| rs11263857 | 1 | 36588402 | A | G | 0.142 | 0.025 | -0.002 | 0.006 |
| rs4074961 | 1 | 38092723 | T | C | -0.100 | 0.015 | 0.007 | 0.006 |
| rs6676847 | 1 | 86666411 | T | C | -0.150 | 0.026 | -0.003 | 0.007 |
| rs12139208 | 1 | 88213014 | C | T | 0.086 | 0.016 | -0.002 | 0.004 |
| rs3737136 | 1 | 113060432 | G | A | -0.113 | 0.019 | -0.002 | 0.004 |
| rs2790052 | 1 | 165738463 | C | G | 0.416 | 0.023 | 0.006 | 0.005 |
| rs1200089 | 1 | 169039605 | T | A | 0.106 | 0.016 | 0.001 | 0.004 |
| rs6691014 | 1 | 219127338 | C | T | 0.200 | 0.030 | 0.000 | 0.007 |
| rs17045881 | 2 | 54649068 | C | G | 0.113 | 0.021 | 0.004 | 0.005 |
| rs1367228 | 2 | 56112440 | A | C | -0.092 | 0.015 | -0.001 | 0.003 |
| rs6732795 | 2 | 69411517 | C | A | -0.095 | 0.016 | 0.001 | 0.004 |
| rs11689168 | 2 | 112621199 | C | T | 0.210 | 0.018 | -0.003 | 0.004 |
| rs1579050 | 2 | 153364527 | G | A | -0.138 | 0.015 | -0.009 | 0.004 |
| rs12466722 | 2 | 205972788 | G | A | 0.122 | 0.018 | -0.004 | 0.004 |
| rs1991161 | 2 | 218676890 | T | C | -0.085 | 0.015 | 0.003 | 0.003 |
| rs11900963 | 2 | 239308049 | T | A | 0.149 | 0.027 | -0.004 | 0.007 |
| rs10510755 | 3 | 50281168 | T | C | -0.135 | 0.024 | -0.008 | 0.006 |
| rs12633969 | 3 | 66859170 | T | G | 0.129 | 0.017 | -0.003 | 0.004 |
| rs13089412 | 3 | 124309917 | C | T | -0.118 | 0.020 | -0.005 | 0.005 |
| rs4955665 | 3 | 169355019 | C | T | 0.104 | 0.016 | 0.004 | 0.004 |
| rs4308305 | 3 | 171831116 | C | T | -0.187 | 0.034 | -0.010 | 0.007 |
| rs9853115 | 3 | 186131600 | A | T | 0.217 | 0.015 | 0.002 | 0.004 |
| rs6787621 | 3 | 188066953 | G | T | -0.111 | 0.017 | 0.006 | 0.004 |
| rs4383619 | 4 | 7863508 | C | T | 0.240 | 0.019 | 0.001 | 0.005 |
| rs368503 | 5 | 14820417 | G | A | 0.109 | 0.017 | 0.003 | 0.004 |
| rs9293289 | 5 | 71683885 | A | G | 0.094 | 0.017 | 0.002 | 0.004 |
| rs990133 | 5 | 108045369 | C | T | 0.129 | 0.023 | -0.006 | 0.005 |
| rs2317961 | 6 | 1533116 | G | A | -0.142 | 0.016 | -0.004 | 0.004 |
| rs1755056 | 6 | 45522660 | A | C | 0.104 | 0.016 | -0.008 | 0.004 |
| rs17752199 | 6 | 51406848 | G | A | 0.196 | 0.025 | 0.004 | 0.005 |
| rs9494457 | 6 | 136474794 | A | T | 0.130 | 0.016 | 0.001 | 0.004 |
| rs9364973 | 6 | 158975716 | T | C | 0.104 | 0.017 | -0.005 | 0.004 |
| rs3012379 | 6 | 170466312 | C | T | 0.153 | 0.023 | 0.006 | 0.005 |
| rs2526099 | 7 | 11677840 | G | A | -0.087 | 0.015 | -0.001 | 0.003 |
| rs10279081 | 7 | 33340516 | G | A | 0.115 | 0.020 | 0.002 | 0.004 |
| rs327719 | 7 | 80839869 | A | G | 0.103 | 0.016 | 0.006 | 0.003 |
| rs6969706 | 7 | 116154831 | T | G | -0.237 | 0.017 | 0.003 | 0.004 |
| rs10505102 | 8 | 108288349 | A | T | 0.245 | 0.022 | 0.004 | 0.005 |
| rs6999582 | 8 | 124558058 | A | G | -0.212 | 0.031 | -0.007 | 0.007 |
| rs6476827 | 9 | 4220832 | G | C | -0.088 | 0.016 | 0.002 | 0.004 |
| rs2472493 | 9 | 107695848 | A | G | 0.220 | 0.015 | -0.001 | 0.004 |
| rs2567707 | 9 | 116930100 | A | G | -0.098 | 0.018 | 0.004 | 0.004 |
| rs1336980 | 9 | 129377855 | C | G | 0.185 | 0.016 | -0.004 | 0.004 |
| rs6586030 | 10 | 82254047 | G | A | -0.132 | 0.021 | 0.002 | 0.005 |
| rs2797569 | 10 | 95044556 | T | G | 0.094 | 0.015 | -0.003 | 0.003 |
| rs1556659 | 10 | 130834698 | T | C | 0.089 | 0.016 | -0.004 | 0.004 |
| rs4963120 | 11 | 825777 | T | C | 0.091 | 0.015 | 0.001 | 0.004 |
| rs4141194 | 11 | 17011176 | A | C | -0.143 | 0.017 | 0.002 | 0.004 |
| rs9666712 | 11 | 44855663 | C | G | 0.142 | 0.026 | 0.007 | 0.006 |
| rs7123436 | 11 | 48013484 | A | G | -0.162 | 0.019 | 0.006 | 0.004 |
| rs1821466 | 11 | 55064357 | C | G | -0.135 | 0.022 | 0.009 | 0.005 |
| rs7939319 | 11 | 86401227 | C | T | -0.137 | 0.019 | -0.003 | 0.004 |
| rs632672 | 11 | 103050028 | G | A | -0.097 | 0.017 | 0.001 | 0.004 |
| rs7102093 | 11 | 115455085 | A | G | -0.093 | 0.017 | -0.001 | 0.004 |
| rs11827818 | 11 | 120198728 | G | A | -0.189 | 0.021 | -0.005 | 0.005 |
| rs7924522 | 11 | 128380742 | A | C | -0.124 | 0.016 | -0.001 | 0.004 |
| rs10860887 | 12 | 102985384 | A | G | -0.089 | 0.016 | -0.007 | 0.004 |
| rs17075835 | 13 | 22666400 | G | A | -0.116 | 0.020 | 0.006 | 0.005 |
| rs1239687 | 13 | 51163561 | G | T | -0.085 | 0.015 | 0.004 | 0.003 |
| rs9544021 | 13 | 76253354 | C | T | -0.098 | 0.015 | 0.000 | 0.003 |
| rs12147852 | 14 | 53361875 | A | G | 0.125 | 0.018 | 0.001 | 0.005 |
| rs9323599 | 14 | 74955679 | C | T | 0.403 | 0.064 | -0.009 | 0.010 |
| rs2249195 | 15 | 61958029 | C | A | 0.110 | 0.015 | 0.000 | 0.003 |
| rs878359 | 16 | 65060817 | G | A | 0.094 | 0.016 | 0.001 | 0.004 |
| rs9033 | 16 | 67181999 | G | A | -0.095 | 0.015 | -0.009 | 0.004 |
| rs12444539 | 16 | 77538003 | T | A | -0.180 | 0.029 | -0.010 | 0.006 |
| rs3743860 | 16 | 89818491 | C | T | 0.104 | 0.015 | -0.001 | 0.004 |
| rs9913911 | 17 | 10031183 | G | A | 0.232 | 0.016 | 0.000 | 0.004 |
| rs10853029 | 17 | 59396449 | T | C | -0.126 | 0.018 | -0.003 | 0.004 |
| rs13042961 | 20 | 11211742 | C | T | 0.217 | 0.038 | -0.010 | 0.009 |
| rs6063517 | 20 | 49059241 | T | A | 0.100 | 0.016 | 0.003 | 0.004 |
| rs17534001 | 22 | 19842310 | A | G | 0.154 | 0.028 | -0.007 | 0.006 |
| rs5997451 | 22 | 29568963 | A | G | -0.180 | 0.028 | 0.007 | 0.007 |
| rs5756813 | 22 | 38175477 | T | G | 0.098 | 0.016 | 0.003 | 0.004 |

**Supplementary Table 4. Mendelian Randomization tests using IOP-associated SNPs from the genetic association analyses in the UK Biobank as instruments over O-Methylascorbate (KORA)**.

| Method | Beta | SE | p-value |
| --- | --- | --- | --- |
| Penalized weighted median | 0.004 | 0.005 | 0.38 |
| Robust inverse-variance weighted | 0.002 | 0.003 | 0.57 |
| Robust MR-Egger | 0.006 | 0.008 | 0.46 |
| Penalized robust MR-Egger (Intercept) | -0.001 | 0.001 | 0.60 |
